# Simvastatin down-regulates the production of Interleukin-8 by neutrophil leukocytes from dyslipidemic patients

**DOI:** 10.1186/1471-2261-14-37

**Published:** 2014-03-15

**Authors:** Franca Marino, Andrea Maria Maresca, Luana Castiglioni, Marco Cosentino, Ramona C Maio, Laura Schembri, Catherine Klersy, Christian Mongiardi, Laura Robustelli Test, Anna Maria Grandi, Luigina Guasti

**Affiliations:** 1 Department of Clinical and Experimental Medicine, University of Insubria, Viale Borri 57, 21100 Varese, Italy; 2 Biometry and Clinical Epidemiology, IRCCS Policlinico S. Matteo, Pavia, Italy

**Keywords:** Neutrophils, Interleukin-8, Dyslipidemia, Statin

## Abstract

**Background:**

Neutrophil (PMN) leukocytes participate to the initial phases of atherosclerosis through the release of Interleukin 8 (CxCL8; IL-8) that contribute to amplification of inflammation. Aim of the study is to investigate the production of IL-8 by PMN leukocytes from dyslipidemic patients treated with simvastatin.

**Methods:**

In 15 dyslipidemic subjects with moderately increased cardiovascular risk, assessed by Framingham Risk Score, blood samples were obtain to investigate PMNs IL-8 production [at baseline and after N-formyl-Met-Leu-Phe (fMLP) stimulation] before and after long-term (1-year) simvastatin treatment.

**Results:**

The resting release of IL-8 was higher in dyslipidemic patients at baseline when compared with control subjects (p < 0.05). One year of treatment was significantly associated with reduced IL-8 production (p < 0.01). Moreover, the fMLP-induced IL-8 production in dyslipidemic untreated patients was higher than that of controls (p < 0.05) and was reduced after simvastatin treatment (p < 0.01). IL-8 release after 1 year of treatment was reduced to levels which were lower than those observed in control subjects both for resting and stimulated cytokine production (p < 0.01).

**Conclusions:**

Prolonged treatment with simvastatin is associated with a reduction of IL-8 production, suggesting the possibility of statin to modulate the pro-inflammatory response in PMNs of patients with moderately increased cardiovascular risk.

## Background

Inflammation plays a key role in the beginning and progression of atherosclerosis [1]. The relation of coronary artery disease with systemic inflammation is confirmed by the closely association between serum markers of inflammation and risk of cardiovascular events [2,3].

Polymorphonuclear leukocytes (PMNs) are effector cells in many inflammatory conditions [4]. These cells produce a large amount of reactive oxygen species, involved in lipid oxidation [5]. In addition, stimulated and activated PMNs release cytokines that produce impairment of endothelial function [6]. On stimulation, PMNs release cytokines such as IL-8 that promote endothelial cell activation and may further affect their recruitment [7]. As regards the functional alterations which occur at the cellular level in atherosclerosis, although a primed state of PMNs - associated with increased oxidative stress, pro-inflammatory cytokine release and increased Angiotensin II type 1 receptor expression - has been described in humans with high cardiovascular risk, hypercholesterolemia, hypertension, diabetes and renal failure, only few data are reported about the potential role of pharmacological treatment on the functional properties of PMNs [8-13]. Previous studies from our group showed that short-term simvastatin treatment was able to affect PMNs functional responses [11-13], however the effect of prolonged statin treatment on IL-8 production has not yet been investigated. Aim of our study was to asses the production of IL-8 by PMN leukocytes from dyslipidemic patients treated with simvastatin.

## Methods

### Subjects

In 15 dyslipidemic patients at moderately increased cardiovascular risk, assessed by Framingham Risk Score [14], who had signed a written informed consent before enrolling in the study, PMNs were isolated from venous blood (see below) before any pharmacological treatment and during short-term (1 month) and long-term (1 year) simvastatin treatment (20 mg assumed at 10 PM) to investigate IL-8 cellular production.

Subjects were enrolled consecutively at our Lipid Clinic (Clinical Medicine, University of Insubria, Varese, Italy).

Inclusion criteria were:

● increased cardiovascular risk showing a “moderate risk” for vascular events according to the National Cholesterol Education Program - Adult Treatment Panel III (ATPIII) guidelines [14]; patients enrolled at “moderate risk” showed in fact two or more risk factors and a 10-year cardiovascular risk < 20% when calculated according to the Framingham algorithm.

● no disease (except dyslipidemia and/or mild hypertension) found after a clinical examination and routine laboratory tests.

● clinical indication of lipid-lowering pharmacological treatment with statins

● no pharmacological treatment.

Exclusion criteria were:

● documented ischemic heart disease or “equivalent ischemic heart disease” [14]

● diabetes

● ongoing clinical infection or presence of infections in the previous three months

● smoking habitus

● competitive sporting activities.

After 6 weeks of life-style modification including dietary treatment (qualitative counselling) and recommendations for mild physical activity, patients were asked to maintain the same level of physical activity and a similar diet throughout the study; besides the evaluations scheduled for the study on neutrophils at 1 month and 1 year, patients were followed for clinical evaluations and standard laboratory exams after starting statin therapy.

A group of 15 healthy subjects selected from a population evaluated for a general clinical check-up was also enrolled: all patients were evaluated with a clinical visit including familiar and personal history.

Blood samplings were obtained to perform routine laboratory exams and to isolate circulating PMNs by using heparinized tubes between 8.00 and 9.00 AM, after a fasting night. All patients were previously asked not to take coffees, teas, chocolates or cola-containing substances for the 24 hours preceding the evaluations.

The following laboratory exams were performed:

● Haemocromocytometric exam, plasma creatinine, urea, aspartate aminotransferase, alanine aminotransferase, gamma glutamyl transpeptidase, alkaline phosphatase, creatine kinase, thyroid stimulating hormone, urinary proteins, plasma protein electrophoresis, fasting glucose: they resulted in the two groups within the normal limits and no clinically relevant change was observed during treatment (data not shown).

● Total cholesterol, high density lipoprotein-cholesterol (HDL-c), low density lipoprotein-cholesterol (LDL-c), triglycerides, apolipoprotein A, apolipoprotein B, high-sensitive C-reactive protein (CRP).

The study protocol was approved by “Azienda Ospedaliera Ospedale di Circolo e Fondazione Macchi” Ethics Committee.

### PMN isolation

Whole blood was allowed to sediment on dextran at 37°C for 30 min. Supernatant was recovered and PMNs were isolated by standard density-gradient centrifugation as previously described [13]. Contaminating erythrocytes were eliminated by 10 minutes hypotonic lysis in distilled water with added NH_4_Cl 8.2 g/l, KHCO_3_ 1.0 g/l, and EDTA 37.0 mg/l. Cells were then washed three times in NaCl 0.15 M. Purity and viability of PMNs preparations were always >95% and no platelets or erythrocytes could be detected either by light microscopic examination or by flow cytometric analysis.

### PMN production of CXCL8

To assess IL-8 production, PMNs were re-suspended at the concentration of 1×10^7^ cells/ml in RPMI medium and incubated alone (resting IL-8 production) or in the presence of 0.1 μM of fMLP (stimulated IL-8 production) at 37°C for 5 hours. After incubation, cells were centrifuged (600 g, 5 min, 20°C) and supernatant was harvested for IL-8 assay. IL-8 levels in PMN supernatants were quantified using a sandwich-type enzyme-linked immunosorbent assay (ELISA kit; Amersham Biosciences, UK). Detection limit of the assay was 1 pg/ml.

Levels of IL-8 were determined in a duplicate way and production of PMNs IL-8 was measured in all patients and controls.

### Statistical analysis

Data are presented as mean ± standard deviation (SD) and median and 25^th^-75^th^ percentile range (IQR), as needed. The levels of IL-8 had not a normal distribution, so we have applied non-parametric tests. Comparisons between dependent measures were performed with the Wilcoxon test. The Bonferroni correction was applied for post-hoc comparisons. Comparisons between independent measures were performed with the MannWhitney U test. The mean difference and its 95% confidence interval (95% CI) was computed to quantify both changes in time and differences between cases and controls. Correlation analysis was performed by Pearson test. Calculations were performed using a commercial software (GraphPad Prism version 4.00 for Windows, GraphPad Software, San Diego, CA, USA, http://www.graphpad.com) and a two-sided *P* < 0.05 was retained for statistical significance.

## Results

Baseline characteristics of dyslipidemic patients and controls are shown in Table 1. The two groups were similar for age, sex, BMI and blood pressure values.

**Table 1:** Clinical and demographic characteristics of dyslipidemic patients and controls at baseline

	**Dyslipidemic patients (n = 15)**	**Controls (n = 15)**	**P**
Age (years)	57 ± 11	53 ± 13	ns
Female (n, %)	8 (53%)	4 (27%)	ns
BMI (kg/m^2^)	26 ± 2	26 ± 2	ns
SBP/DBP (mmHg)	135 ± 14/83 ± 7	122 ± 35/83 ± 8	ns
10 years-cardiovascular risk (%)	9.5 ± 2.1	3.3 ± 1.8	0.01

BMI: body mass index; SBP: Systolic blood pressure; DBP: Diastolic blood pressure.

No patients were lost to the follow up.

### IL-8 production from PMNs of dyslipidemic patients and controls

**Figure 1:**
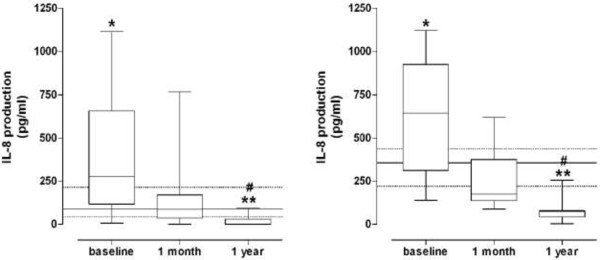
**IL-8 production in resting (left panel) and fMLP-induced (right panel) PMNs from dyslipidemic subjects before (baseline) and during statin treatment.** Horizontal lines represent the median values, boxes represent the 25^th^-75^th^ percentiles, and vertical bars the minimum and maximum values. For comparison, data obtained in healthy controls are also shown: median values (solid lines) with 25^th^-75^th^ percentiles (dotted lines). * = *P* < 0.05 and ** = *P* < 0.01 *vs* control subjects. # = *P* < 0.01 *vs* baseline.

The resting release of IL-8 was different between controls and dyslipidemic subjects at baseline [mean difference (95% C.I.): −280.4 pg/ml (IQR: −454.0 to −106.8), p < 0.05]. One year of treatment significantly reduced IL-8 production [mean difference (95% C.I.): 399.5 pg/ml (IQR: 187.7 to 611.3)]. IL-8 production after 1 year of treatment was reduced to levels which were lower than those observed in control subjects [mean difference (95% C.I.): 119.1 pg/ml (IQR: 66.8 to 171.4)] (Figure 1, left panel).

Moreover, the fMLP-induced IL-8 production in untreated dyslipidemic patients was higher than that of controls [mean difference (95% C.I.): −263.4 pg/ml (IQR: −469.0 to −57.91), p < 0.05]. The stimulated IL-8 production was reduced after simvastatin treatment [mean difference (95% C.I.): 543.9 pg/ml (IQR: 356.8 to 731.0)]. The values measured at 1-year follow-up were lower than those observed in control subjects [mean difference (95% C.I.): 280.5 pg/ml (IQR: 170.8 to 390.1)] (Figure 1, right panel).

Basal levels of IL-8 didn’t correlate to age (r = 0.463, P = ns), CRP (r = 0.341, P = ns), non-HDL cholesterol (r = 0.317, P = ns) and apoB/apoA ratio (r = 0.301, P = ns).

We didn’t found a significant correlation between IL-8 reduction (from baseline to 1 year) and changes of other parameters (LDL-cholesterol, CRP).

### Clinical and lipid profile changes during statin treatment in dyslipidemic patients

Body mass index and blood pressure values did not significantly change during follow-up. The lipid profile of patients and controls at baseline and after 1 year is shown in Table 2. As expected, in dyslipidemic patients, total cholesterol, LDL-c and apolipoprotein B were significantly reduced at 1-year evaluation compared to baseline evaluation [total cholesterol: 204 mg/dL (IQR: 171–232) vs 276 mg/dL (IQR: 256–330), *P* < 0.0001; LDLc: 118 mg/dL (IQR: 103–149) vs 202 mg/dL (IQR: 191–246), *P* < 0.0001; apolipoprotein B: 100 mg/dL (IQR: 86–119) vs 164 mg/dL (IQR: 157–219), *P* = 0.0001]. HDL-c, triglycerides and apolipoprotein A did not significantly change during follow-up. We performed a subgroup analysis by gender (Table 3) and we observed a major reduction of LDL-cholesterol in women. Moreover, CRP values was significantly reduced during treatment [2.99 mg/L (IQR: 1.33-4.86) and 1.5 mg/L (IQR: 1.0-2.6), *P* < 0.01)].

**Table 2:** Lipid profile of dyslipidemic patients and controls at baseline and after 1 year

	**Dyslipidemic patients (n = 15)**		**Controls (n = 15)**	
	**Baseline**	**After 1 year**	**Baseline**	**After 1 year**
Total cholesterol (mg/dl)	276 (256–330)	204 (171–232)*	245 (188–266)°	188 (165–200)
HDL-cholesterol (mg/dl)	50 (47–57)	52 (49–65)	56 (47–63)	53 (46–65)
LDL-cholesterol (mg/dl)	202 (191–246)	118 (103–149)*	163 (116–175)°	112 (87–122)
Triglycerides (mg/dl)	141 (115–190)	144 (87–154)	159 (74–198)	133 (84–160)
Apoliprotein B (mg/dl)	164 (157–219)	100 (86–119)*	140 (119–157)°	149 (128–185)
Apoliprotein A (mg/dl)	143 (135–162)	141 (136–175)	105 (91–146)	99 (77–116)

*vs baseline (p < 0.0001).

°vs dyslipidemic patients at baseline (p < 0.001).

**Table 3:** Modification in lipid profile of dyslipidemic patients after simvastatin treatment by gender

	**Male (n = 7)**		**Female (n = 8)**	
	**Baseline**	**After treatment**	**Baseline**	**After treatment**
Total cholesterol (mg/dl)	260 (236–316)	208 (172–263)	299 (278–347)	199 (176–229)*
LDL-cholesterol (mg/dl)	186 (172–207)	120 (99–160)^**+**^	207 (187–244)	118 (142–203)*
HDL-cholesterol (mg/dl)	50 (47–57)	54 (48–70)	51 (47–60)	52 (49–63)
Tryglicerides (mg/dl)	115 (87–134)	114 (83–167)	180 (142–203)	149 (95–153)
Apolipoprotein A (mg/dl)	141 (136–152)	136 (127–166)	145 (134–163)	147 (136–177)
Apolipoprotein B (mg/dl)	151 (139–168)	90 (85–126)^+^	180 (136–269)	101 (91–119)*

*vs baseline (p = 0.001).

+vs baseline (p = 0.05).

## Discussion

It is clear that atherosclerosis is a chronic disease of arterial wall in which immuno-inflammatory mechanisms are involved [15]. It is well known the role of monocytes, as representatives of the innate immune system, in atherosclerosis development [16]. Since a body of research carried out over the last decade has disclosed the complex behaviour of PMNs, unraveling a key role in the onset and progression of atheroma, we decided to focus our attention on these cells [17].

The main finding of this study is the observation that a prolonged treatment with statin, in patients with increased cardiovascular risk, is consistently associated with reduction in IL-8 cellular production by primed neutrophils. Pathogenic effects of PMNs in atherosclerosis are mediated through production of pro-inflammatory cytokines [IL-8, tumor necrosis factor alpha (TNFa)] and reactive oxygen species [18]. Chemokines are a number of small, inducible, proinflammatory proteins that direct migration of circulating leukocytes to sites of inflammation. This superfamily is divided into α and β-chemokine subfamilies. IL-8 is one of the main proinflammatory cytokine produced by neutrophils and it is the prototypical member of α-chemokine subfamily [19-24]. Generation of IL-8 can be expected upon infection, ischemia, trauma and other disturbances of tissue homeostasis since the levels of IL-1 and TNFa, which are important IL-8 inducers, are elevated. IL-8 is likely to be the main cause of local accumulation of neutrophils [20]. IL-8 might have atherogenic function through multiple actions: it facilitates recruitment of neutrophils and T-lymphocytes into sub-endothelial space, monocytes activation and adhesion to endothelium [21,22], migration of vascular smooth muscle cells [22]. Macrophage-derivated human foam cells contain high amounts of IL-8 [23,24]. It has also been reported that apparently healthy subjects in the highest serum IL-8 level quartile had an increased risk of coronary events when compared with those in the lowest quartile [25].

In our study we observed a lack of significant correlation between basal levels of IL-8 and age, CRP, non HDL cholesterol and apoB/apoA ratio. Furthermore despite a similar reduction after simvastatin treatment (from baseline to 1 year) of IL-8, LDL cholesterol and CRP, we did not see any significant correlation. From our point of view these results are largely explained by the small sample size of the study.

Statins have pharmacological activities independently of their lipid-lowering action, that can be in part attributed to their ability to interfere with inflammatory mechanisms [26]. Among the several ancillary actions of statins, these drugs are able to down-regulate expression of adhesion molecules, to inhibit expression of chemokine monocyte chemo-attractant protein-1 in activated leukocytes, to block expression of integrins and to reduce monocytes adhesiveness to endothelium [27-30]. As regards IL-8 production by human neutrophils, it has been demonstrated that simvastatin treatment could reduce peripheral blood monocyte mRNA expression of IL-8 and IL-6 and monocyte chemo-attractant protein-1 after 6 week of treatment [31] and we reported that cellular PMNs IL-8 release was reduced upon 1-month statin therapy [12]. In this study, one year of simvastatin treatment reduced IL-8 production in comparison to pre-treatment values, thus excluding the occurrence of a potential tolerance phenomenon after long-term treatment. Moreover, IL-8 production after 1 year of treatment was reduced to levels which were lower than those observed in control subjects. Our results seem to suggest that simvastatin can contribute to modulate inflammation by IL-8 reduction.

In our study simvastatin treatment reduced LDL cholesterol in men and in women, but the degree of this lowering was more evident in females. This result may be in part due to a higher LDL cholesterol baseline value than in men and to the prevalent menopausal status in enrolled women. Nevertheless the small sample size (8 women) does not allow to draw definitive conclusions. From recent meta-analyses statin therapy is associated with significant decreases in cardiovascular events and in all-cause mortality in women and men [32].

The study has some limitations: 1) we have not collected informations about subclinical atherosclerosis (intima-media thickness, carotid plaque); 2) we have evaluated IL-8 production as marker of inflammatory response, but inflammation can also be evaluated by several different parameters, such as IL-6, IL-15, TNFα, not assessed in this study.

## Conclusions

Our results show an enhanced production of IL-8 in patients at moderately increased risk for vascular disease. Simvastatin treatment is associated with a reduced production of IL-8 from PMNs in a long-time treatment.

## Abbreviations

PMNs: Neutrophils, polymorphonuclear leukocytes; IL-8: CXCL8, interleukin-8; fMLP: N-formyl-Met-Leu-Phe; ATPIII: Adult Treatment Panel III; HDL-c: High density lipoprotein-cholesterol; LDL-c: Low density lipoprotein-cholesterol; CRP: C-reactive protein; TNFa: Tumor necrosis factor alpha.

## Competing interests

The authors declare that they have no competing interests.

## Authors’ contributions

FM, AMM and LG: design, data collection, drawing the manuscript, data analysis and statistics. All authors: design, critical revision of article and approval of article.

## Pre-publication history

The pre-publication history for this paper can be accessed here:

http://www.biomedcentral.com/1471-2261/14/37/prepub
